# Respiratory morbidity in preterm infants predicted by natriuretic peptide (MR-proANP) and endothelin-1 (CT-proET-1)

**DOI:** 10.1038/s41390-021-01493-8

**Published:** 2021-05-06

**Authors:** Roland Gerull, Roland P. Neumann, Andrew Atkinson, Luca Bernasconi, Sven M. Schulzke, Sven Wellmann

**Affiliations:** 1 Department of Neonatology, University Children’s Hospital Basel UKBB, University of Basel, Basel, Switzerland; 2 Division of Neonatology, University Children’s Hospital Inselspital Berne, Berne, Switzerland; 3 Department of Pediatric Pharmacology, University Children’s Hospital Basel UKBB, University of Basel, Basel, Switzerland; 4 Institute for Laboratory Medicine, Kantonsspital Aarau, Aarau, Switzerland; 5 Department of Neonatology, University Children’s Hospital Regensburg (KUNO), University of Regensburg, Regensburg, Germany

## Abstract

**Background:**

Bronchopulmonary dysplasia (BPD) is a major complication in preterm infants <32 weeks. We aimed to assess whether plasma levels of mid-regional pro-atrial natriuretic peptide (MR-proANP) and C-terminal pro-endothelin-1 (CT-proET-1) predict respiratory morbidity.

**Methods:**

This was a prospective, two-center, observational cohort study. MR-proANP and CT-proET-1 were measured at day 7 (±2) of life. Associations with duration of supplemental oxygen and the composite outcome of moderate or severe BPD or death (BPD/death) were investigated.

**Results:**

Two hundred and twenty-nine infants <32 weeks were included (median gestational age [GA] 29.6 weeks [interquartile range 29.0–30.7], median birth weight 1150 g [IQR 840–1410]). MR-proANP and CT-proET-1 were associated with the duration of supplemental oxygen in univariable analysis (both *p* < 0.001) but not after adjusting for co-factors. Infants with BPD/death showed higher plasma levels of MR-proANP (623.50 pmol/L [IQR 458.50–881.38] vs. 308.35 pmol/L [IQR 216.72–538.10]; *p* < 0.001) and CT-proET-1 (255.40 pmol/L [IQR 202.60–311.15] vs. 198.30 pmol/L [IQR 154.70–297.95]; *p* = 0.015) compared to infants without BPD/death. Levels of both biomarkers were significantly associated with BPD/death in univariable models but not after adjusting for co-factors.

**Conclusions:**

MR-proANP and CT-proET-1 are associated with the duration of supplemental oxygen and the composite outcome BPD/death, but their prognostic value does not complement that of clinical risk factors.

**Impact:**

- Plasma levels of MR-proANP and CT-proET-1, measured on day 7 of life (±2 days) are associated in univariable analyses with duration of supplemental oxygen and the combined outcome of BPD or death in VLGA infants.
- Associations between both biomarkers and respiratory morbidity do not persist in multivariable models, in particular when gestational age is included.
- MR-proANP and CT-proET-1 have limited additional value to predict respiratory morbidity in VLGA infants compared to clinical parameters.

## Introduction

Despite advances in neonatal intensive care, bronchopulmonary dysplasia (BPD) continues to be one of the major complications of preterm birth. Unlike other neonatal morbidities, the rate of BPD has remained relatively stable over the past decades, most likely due to the improved survival of extremely preterm infants.^1–3^ The burden of disease is extensive, since BPD is not only associated with increased long-term respiratory morbidity^4,5^ but may also result in increased risks for cardiovascular morbidity, growth, and neurodevelopmental impairment as well as reduced quality of life.^4,6,7^ Once BPD is established, treatment options are limited and are symptomatic rather than curative.^8^ Therefore, early identification of infants at risk for BPD development is crucial in order to prevent sequelae of the disease. The pathogenesis of BPD is complex and includes lung injury, alveolar growth arrest, inflammation, oxygen toxicity, and others, which makes it challenging to find reliable predictors. Numerous prediction tools with modest positive and negative predictive values have been developed, indicating that parameters that are more reliable are urgently needed.^9,10^

Several biomarkers have been studied as predictors for BPD development in preterm infants, including the vasoactive peptides brain-type natriuretic peptide (BNP) and endothelin-1 (ET-1). BNP measured on day of life (DOL) 3, as well as at the age of 4 weeks, has been previously shown to be associated with subsequent development of BPD.^11,12^ Atrial natriuretic peptide (ANP) belongs, like BNP, to the family of natriuretic peptides and is released from atrial myocytes in response to stretching of the atrial wall.^13^ Both natriuretic peptides are believed to bind at the same receptor.^14^ Elevated levels of ANP predict death and serious cardiovascular events in adults.^15^ However, its role as predictor of respiratory morbidity in preterm infants is largely unknown.

ET-1 acts as potent vasoconstrictor and can induce vascular remodeling as well as fibrosis,^16^ which are both features of BPD. In a previous study, we showed that plasma C-terminal pro-endothelin-1 (CT-proET-1) was associated with the duration of supplemental oxygen and the diagnosis of BPD, when measured at the end of the first week of life.^17^ However, since three different cohorts provided data on CT-proET-1 measurements in a cross-sectional manner, results were therefore suggestive but not conclusive.

Based on the association between early measured CT-proET-1 and respiratory morbidity, we conducted a prospective study to assess the role of mid-regional pro-atrial natriuretic peptide (MR-proANP) and CT-proET-1 as predictors for respiratory morbidity in very low gestational age (VLGA) newborns. We hypothesized that plasma levels of MR-proANP and CT-proET-1, measured on DOL 7, are associated with (i) the duration of supplemental oxygen and (ii) the development of the composite outcome of BPD/death. Furthermore, we hypothesized that (iii) these biomarkers provide additional benefit for the prediction of BPD compared to solely clinical parameters.

## Methods

### Study design

We conducted a prospective, observational, two-center cohort study (Clinical Trials Identifier: NCT02083562) at the Department of Neonatology, University Children’s Hospital Basel UKBB and University Children’s Hospital Berne, Switzerland. Both are referral centers for high-risk infants and provide tertiary-level neonatal care. We enrolled participating infants into the study after obtaining written informed parental consent. The Ethics Committee of Northwestern and Central Switzerland (Basel, study No. 233/13) and the Ethics Commission of the Canton of Berne (Berne, study No. 2481) approved the study.

### Associations and outcomes

We assessed the association between plasma levels of biomarkers (MR-proANP and CT-proET-1) on DOL 7 (±2 days) with the primary outcome of duration of supplemental oxygen measured between birth and hospital discharge. We further analyzed the association of levels of plasma biomarkers (MR-proANP and CT-proET-1) on DOL 7 (±2 days) with the secondary composite outcome of BPD (defined as moderate or severe BPD according to the NHBLI workshop definition^18^) or death (BPD/death). At 36 weeks post-menstrual age (PMA), we performed a standardized BPD assessment.^19^ In additional analyses, we assessed whether biomarkers provided additional value for BPD/death prediction compared to clinical parameters in (i) univariable analysis, (ii) multivariable analyses including risk factors identified by univariable analyses, or (iii) multivariable analyses including parameters of the National Institute of Child Health and Development (NICHD) Neonatal Research Network BPD risk estimator.^20^ We also analyzed these outcomes in a subset of patients with a GA of <27 weeks.

Duration of supplemental oxygen and mechanical ventilation were determined as hours from birth until hospital discharge. On the day of blood sampling, we also collected data on supplemental oxygen and type of ventilator support.

Clinical parameters were prospectively recorded and completed at patient discharge from patient charts. The following definitions were applied: small for GA: birth weight <10th percentile; antenatal corticosteroids: any corticosteroids given for lung maturation; chorioamnionitis: clinical or histological proof of infection; sepsis: clinical manifestation of systemic infection, including but not limited to positive blood culture; patent ductus arteriosus: hemodynamically relevant, as classified by the cardiologist performing the echocardiography; intraventricular hemorrhage (IVH) >II° as defined by Papile et al.^21^; total respiratory support: combined time of mechanical ventilation, non-invasive positive pressure ventilation, continuous positive airway pressure, and high flow nasal cannula.

### Patients

Preterm infants <32 weeks of GA (VLGA) were eligible for this study. Exclusion criteria were the absence of parental consent and the presence of major congenital malformations, including relevant congenital heart defects and primary pulmonary malformations. Patients were recruited consecutively between November 2013 and November 2017. Based on researchers’ availability, all parents or legal guardians of VLGA infants meeting the inclusion criteria were approached.

### Biomarker analysis

We took samples of 500 µL blood in EDTA microtubules on DOL 7 (±2 days) in participating infants and centrifuged the sample within 2 h, and froze the plasma at −80 °C. BRAHMS KRYPTOR analyzed the levels of MR-proANP and CT-proET-1 in three batches with automated immunofluorescent assays (BRAHMS Biomarkers, Thermo Scientific, Henningsdorf, Germany). Both fully automated assays are based on the sandwich immunoassay technique described in detail elsewhere.^22,23^

### Sample size calculation

Based on results from a previous study on CT-proET-1 in VLGA newborns,^17^ a minimum number of 210 neonates was required to obtain a statistically significant regression coefficient at a level of significance of *p* < 0.05 with at least 90% power. This calculation was based on a multivariable linear regression model including duration of supplemental oxygen as the dependent variable, which was adequately transformed to achieve normality, and CT-proET-1 levels as the independent variable, taking also into account confounders, such as sex, GA, and sepsis. Considering a 10% rate of analytical or pre-analytical dropouts, we aimed for inclusion of 231 VLGA patients for this study.

### Statistical analyses

Results for the continuous variables are reported as median and interquartile ranges [IQRs], and categorical variables are expressed as absolute frequency with corresponding percentage. We compared groups for continuous and categorical variables by Wilcoxon–Mann–Whitney test and Chi-square tests, respectively.

We investigated associations using univariable and multivariable linear models with log_10_ or Box–Cox transformed duration of supplemental oxygen as dependent variable and similarly transformed biomarker values (MR-proANP and CT-proET-1) as independent variables, along with the following clinical parameters, which are known to influence respiratory morbidity in preterm infants: GA, birth weight *z*-score, ethnicity, sex, type of respiratory support, FiO_2_, duration of mechanical ventilation, and sepsis. To determine the most parsimonious multivariate model, we included those variables significant at the 5% level in univariate analysis and then used forwards and backwards variable selection with the Bayesian Information Criteria as inclusion/exclusion metric. We estimated risk factors for BPD/death estimated by fitting univariable and multivariable logistic regression models with BPD/death defined at 36 weeks (no/yes) as dependent variable, and log_10_ or Box–Cox transformed biomarker values (MR-proANP and CT-proET-1), along with GA, birth weight *z*-score, ethnicity, FiO_2_, type of respiratory support, duration of mechanical ventilation, and sepsis as independent variables. The final model was again identified by including those covariates significant at the 5% level from univariate analysis and subsequently using forwards and backwards selection.

We investigated the predictive power of the univariable models with BPD/death as dependent variable and the biomarkers as independent variable (and in combination) using receiver operating characteristic (ROC) curves. A standard *N*-fold cross-validation technique was implemented in which the data set was bootstrapped and then partitioned into training and test data sets by leaving out *X* of the total *M* records. The respective logistic model was fit to the (*M* *−* *X* record) data sets (i.e., training) and then the resulting model tested using the “left-out” *X* data records (i.e., test). We calculated the ROC curves based on the predictive results and repeated this process for different values of *X* to test sensitivity to the number of folds.

Furthermore, we analyzed whether biomarkers improved the NICHD BPD risk estimator, when added either individually or in combination to the seven parameters (GA, birth weight, sex, ethnicity, postnatal day, ventilator type, and FiO_2_) used in the risk estimator. We compared the area under the ROC curve (AUC) from the resulting plots with associated 95% confidence intervals (CIs) calculated using the DeLong approach. Throughout, a *p* value of <0.05 was considered statistically significant. We performed statistical analyses in R (R: A Language and Environment for Statistical Computing; version 3.6.1; R Core Team 2019).

## Results

**Fig. 1:**
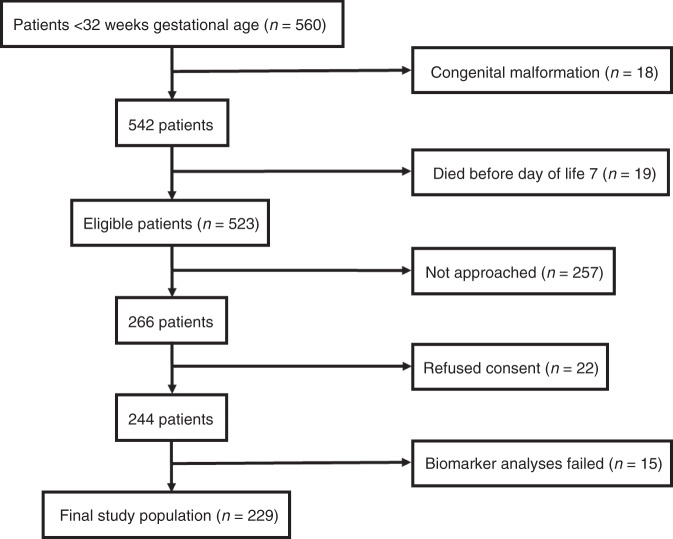
Flow chart of patient recruitment.

This study included 229 preterm infants with a median [IQR] GA and birth weight of 29.6 weeks [27.0–30.7] and 1150 g [840–1410], respectively. Figure 1 shows a flow chart of patient recruitment; Table 1 provides detailed patient characteristics.

**Table 1:** Patient characteristics, levels of biomarkers, and respiratory morbidity.

	All (*n* = 229)	No BPD/death (*n* = 203)	BPD/death (*n* = 26)	*p* value^a^
*Gestational age (weeks)*				
Median	29.57	29.86	25.50	<0.001
[IQR]	[27.00, 30.71]	[27.79, 31.00]	[24.43, 26.29]	
Range	23.71–31.85	23.71–31.85	23.71–30.14	
*Birth weight (g)*				
Median	1150 g	1200 g	650 g	<0.001
[IQR]	[840, 1410]	[910, 1450]	[540, 778]	
Range	370–2440 g	400–2440 g	370–1490 g	
*Ethnicity, n (%)*				
White	220 (96.1)	197 (97.0)	23 (88.5)	0.002
Hispanic	4 (1.7)	4 (2.0)	0 (0.0)	
Black	5 (2.2)	2 (1.0)	3 (11.5)	
*Blood sampling (day of life)*				
Median	7	7	7	0.02
[IQR]	[7, 7]	[7, 7]	[7, 8]	
*Type or respiratory support*^b^*, n (%)*				
Mechanical ventilation	23 (10.0)	12 (5.9)	11 (42.3)	<0.001
CPAP	104 (45.4)	92 (45.3)	12 (46.2)	
None	101 (44.1)	99 (48.8)	2 (7.7)	
*FiO*_*2*_^b^				
Median	0.21	0.21	0.25	<0.001
[IQR]	[0.21, 0.21]	[0.21, 0.21]	[0.22, 0.30]	
SGA, *n* (%)	23 (10.0)	15 (7.4)	8 (30.8)	0.001
Male, *n* (%)	119 (52.0)	108 (53.2)	11 (42.3)	0.402
Any antenatal steroids, *n* (%)	178 (77.8)	159 (78.3)	19 (73.1)	0.685
Chorioamnionitis, *n* (%)	51 (22.3)	43 (21.2)	8 (30.8)	0.392
Preeclampsia, *n* (%)	33 (14.4)	28 (13.8)	5 (19.2)	0.457
Any surfactant, *n* (%)	123 (53.7)	101 (49.8)	23 (84.6)	0.002
Sepsis, *n* (%)	42 (18.3)	27 (13.3)	15 (57.7)	<0.001
PDA day 7, *n* (%)	50 (21.8)	37 (18.2)	13 (50.0)	0.001
IVH, *n* (%)	26 (11.4)	23 (11.3)	3 (11.5)	1.000
Death, *n* (%)	3 (1.3)	0 (0.0)	3 (11.5)	<0.001
*MR-proANP (pmol/L)*				
Median	341.50	308.35	623.50	<0.001
[IQR]	[218.50, 588.82]	[216.72, 538.10]	[458.50, 881.38]	
*CT-proET-1 (pmol/L)*				
Median	202.80	198.30	255.40	0.015
[IQR]	[159.25, 301.30]	[154.70, 297.95]	[202.60, 311.15]	
*O*_*2*_ *supplementation (h)*				
Median	72.0	43.0	2103.9	<0.001
[IQR]	[2.5, 726.0]	[0.75, 338.9]	[1536.4, 2556.0]	
*Mechanical ventilation (h)*				
Median	6.0	1.0	412.4	<0.001
[IQR]	[0.0, 47.3]	[0.0, 22.0]	[111.4, 769.9]	
*CPAP (h)*				
Median	414.3	164.5	938.5	<0.001
[IQR]	[51.0, 740.0]	[50.0, 601.0]	[696.5, 1337.5]	
*High flow nasal cannula (h)*				
Median	160.3	0.0	166.3	<0.001
[IQR]	[0.0, 236.5]	[0.0, 177.0]	[0.0, 424.5]	
*Total respiratory support (h)*				
Median	547.5	341.0	1959.0	<0.001
[IQR]	[71.0, 1089.0]	[60.5, 941.9]	[969.8, 2466.8]	

Chorioamnionitis: clinical or histological signs of infection, PDA: classified by the cardiologist as hemodynamically relevant, total respiratory support: combined duration of mechanical ventilation, non-invasive ventilation, CPAP, and high flow nasal cannula.

*BPD* bronchopulmonary dysplasia, *IQR* interquartile range, *SGA* small for gestational age (birth weight <10th percentile), *PDA* patent ductus arteriosus, *IVH* intraventricular hemorrhage (>grade II), *MV* mechanical ventilation.

^a^*p* Values refer to comparisons of patients “No BPD/death” and “BPD/death.”

^b^On the day of blood sampling.

**Fig. 2:**
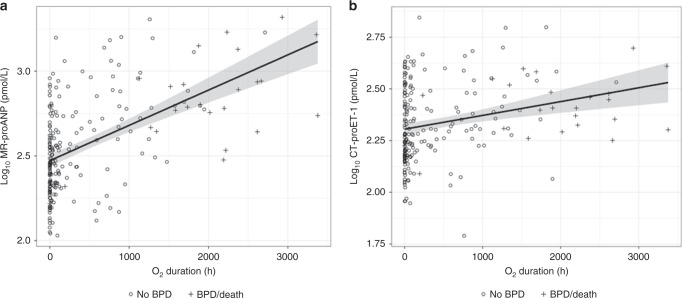
Association of biomarkers with the duration of supplemental oxygen. **a** Association of Log10 transformed levels of MR-proANP with duration of supplemental oxygen. **b** Association of Log10 transformed levels of CT-proET-1 with duration of supplemental oxygen. Circles represent patients without development of BPD/death; crosses show patients with BPD/death.

Median [IQR] plasma levels of MR-proANP and CT-proET-1 on DOL 7 (±2 days) were 341.50 pmol/L [218.50–588.82] and 202.80 pmol/L [159.25–301.30], respectively. Median [IQR] duration of supplemental oxygen was 72.0 h [2.5, 726.0]. Plasma levels of MR-proANP and CT-proET-1 were positively associated in univariable analysis with the duration of supplemental oxygen (both *p* < 0.001; Fig. 2).

Overall, 26 (11.3%) patients met the secondary outcome criterion of BPD/death (Table 1). These infants were of lower GA and birth weight than patients not developing BPD/death. Univariable analyses revealed that patients with BPD/death showed significantly higher median [IQR] plasma levels of MR-proANP and CT-proET-1 (623.50 pmol/L [458.50–881.38] vs. 308.35 pmol/L [216.72–538.10]; *p* < 0.001) and 198.30 pmol/L [154.70–297.95] vs. 255.40 pmol/L [202.60–311.15]; *p* = 0.015, respectively). Both biomarkers were negatively correlated with GA (MR-proANP *p* < 0.001, CT-proET-1 *p* < 0.001).

GA showed a predictive value for development of BPD/death with AUC 0.85 [95% CI 0.77, 0.93] (Fig. 2a), which was superior to that of MR-proANP (AUC 0.76 [0.65, 0.86]) and CT-proET-1 (AUC 0.61 [0.51, 0.72]).

Biomarkers were not associated with BPD/death after adjusting for risk factors identified by univariable analyses (GA, birth weight *z*-score, FiO_2_, sepsis; Table 2). This was true for each of the two biomarkers and the combination of both biomarkers.

**Table 2:** Univariable and fully adjusted multivariable estimates of association between biomarkers and clinical parameters with BPD/death.

BPD/death (yes/no)	Univariable		Multivariable	
	OR [95% CI]	*p* value	OR [95% CI]	*p* value
MR-proANP^a^ (pmol/L)	3.00 [1.83, 5.25]	<0.001	—	nS
CT-proET-1^a^ (pmol/L)	1.63 [1.06, 2.57]	0.03	—	nS
GA (days)	0.50 [0.38, 0.62]	<0.001	0.66 [0.47, 0.90]	0.01
Birth weight *z*-score	0.52 [0.32, 0.83]	0.007	0.49 [0.24, 0.93]	0.03
*Sex*				
Female	1 (reference)	0.3		nS
Male	0.65 [0.28, 1.46]			
*Ethnicity*				
White	1 (reference)	0.05		nS
Non-White	4.28 [0.98, 17.43]			
*Type of respiratory support*^b^				
None	1 (reference)	0.001		nS
Mechanical ventilation	45.38 [10.66, 317.50]	0.02		
CPAP	6.46 [1.70, 42.21]			
FiO_2_^b^	1.29 [1.17, 1.46]	<0.001	1.14 [1.02, 1.30]	0.03
Mechanical ventilation (h)	1.01 [1.00, 1.01]	<0.001	—	nS
*Sepsis*				
No	1 (reference)		1 (reference)	
Yes	9.78 [4.04, 24.69]	<0.001	7.76 [2.42, 28.0]	<0.001

Sepsis: clinical manifestation of systemic infection, including but not limited to positive blood culture.

*GA* gestational age, *nS* non-significant, *OR* odds ratio.

^a^Box–Cox transformation to achieve approximate normality.

^b^On the day of blood sampling.

**Fig. 3:**
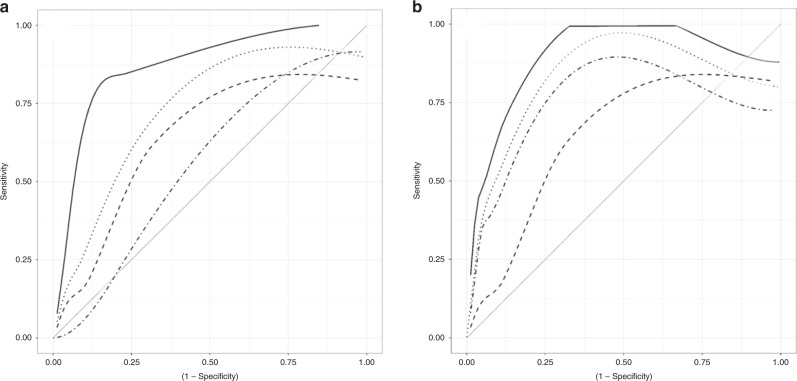
ROC curves for the fitted logistic regression models with BPD/death as dependent variable. **a** Independent variables are gestational age (solid, AUC 0.85 (95% CI [0.77, 0.93])), MR-proANP (dotted, AUC 0.76 [0.65, 0.86]), CT-proET-1 (dot-dashed, AUC 0.61 [0.51, 0.72]), and a composite variable of MR-proANP and CT-proET-1 (dashed, AUC 0.72 [0.61, 0.83]). **b** Independent variables are those included in the NICHD BPD risk estimator (solid, AUC 0.86 [0.76, 0.95]), composite variable of NICHD BPD risk estimator variables plus MR-proANP (dotted 0.84 [0.72, 0.95]), composite variable of NICHD BPD risk estimator variables plus CT-proET-1 (dot-dashed, AUC 0.82 [0.69, 0.95]), and composite variable of NICHD plus MR-proANP plus CT-proET-1 (dashed, AUC 0.83 [0.71, 0.95]). AUC area under the curve.

Similarly, MR-proANP and CT-proET-1 did not improve BPD prediction compared to the multivariable model of the NICHD BPD risk estimator (GA, birth weight, sex, ethnicity, postnatal day, ventilator type, FiO_2_). This was true for inclusion of either one or both biomarkers in the multivariate models (Fig. 3b).

Subgroup analyses of patients with high risk for BPD development (<27 weeks GA, *n* = 56) showed similar results, without additional value of MR-proANP or CT-proET-1.

## Discussion

To our knowledge, this is the largest prospective study assessing MR-proANP and CT-proET-1 as markers for subsequent respiratory morbidity in VLGA infants. Results indicate that levels of both biomarkers, measured on DOL 7, are associated in univariable analysis with the duration of supplemental oxygen and diagnosis of BPD/death at 36 weeks PMA. Plasma levels of MR-proANP showed better predictive values than those of CT-proET-1. However, associations of biomarker levels with supplemental oxygen and BPD/death did not persist in multivariable models, in particular when GA was included. Furthermore, both biomarkers did not improve BPD prediction when combined with clinical risk factors or variables used in the NICHD BPD risk estimator.

Stressed and distended atrial myocytes release ANP in response to volume and pressure load. Since pulmonary hyperperfusion and elevated pulmonary pressures are risk factors for respiratory morbidity, it is biologically plausible that elevated ANP levels may predict BPD development. Whereas ANP is unstable, and therefore unsuited for diagnostic use, its secretion can be estimated by measuring the mid-regional portion of the ANP precursor pro-natriuretic peptide (MR-proANP). While previous studies evaluating natriuretic peptides primarily focused on BNP, ANP has not been studied as a marker of respiratory morbidity in preterm infants. However, studies in children and adults suggest that the diagnostic utility of BNP and ANP in terms of predicting cardiac failure is expected to be similar.^24–26^ To our knowledge, no other prospective studies on the association between ANP and respiratory morbidity in preterm infants have been published.

Previous studies assessing BNP in preterm infants showed moderate associations with pulmonary hypertension^27,28^ and BPD development.^11,12,29,30^ A recent systematic review of observational studies assessed NT-proBNP as an early marker for respiratory morbidity in preterm infants.^29^ Only two small studies with considerable risk of bias were included, thus quality of evidence was deemed to be low. For BPD prediction, sensitivity was 0.86 [95% CI 0.57, 0.98] and specificity was 0.76 [95% CI 0.61, 0.87]. One additional prospective study assessed BPD prediction by measuring NT-proBNP on DOL 28 and included 70 patients.^31^ Patients who subsequently developed moderate or severe BPD showed higher plasma levels of NT-proBNP. In light of these results, our study adds further evidence that increased levels of natriuretic peptides measured at the end of the first week of life are associated in univariable analyses with subsequent development of BPD.

All three known isoforms of endothelin (ET-1, ET-2, and ET-3) are involved in vascular function.^32^ ET-1 is the dominant isoform and therefore most frequently studied. It has a long-lasting vasoconstrictive effect and is involved in vascular remodeling, fibrosis, cell proliferation, and apoptosis.^16,33^ Since pulmonary vasoconstriction and pulmonary hypertension are associated with BPD development, ET-1 is likely to be involved and might have a role as an early marker of BPD development. Since ET-1 is unstable, it is possible to estimate plasma levels by measuring the CT-proET-1.

Clinical studies have shown that ET-1 concentrations were significantly higher in infants with respiratory distress syndrome (RDS) than controls. Among infants with RDS, ET-1 was significantly higher in those who later developed BPD.^34^ Furthermore, ET-1 was significantly elevated in tracheal aspirates of preterm infants on DOL 1, 3, and 7 who subsequently developed BPD.^35^ Only few studies evaluated CT-proET-1 for BPD development. A previous study from our group included 227 VLGA infants and we measured plasma CT-proET-1 at birth, DOL 2, 3, 6, and 28.^17^ This study had a retrospective design; included patients did not participate in the presented prospective study, thus there was no overlap of study participants. The retrospective study showed that predictive value of CT-proET-1 for BPD development was poor at birth but was excellent on DOL 6. Furthermore, CT-proET-1 levels at DOL 2, 3, 6, and 28 were strongly related to the duration of oxygen supplementation. The differences of BPD prediction between the previous study and the presented prospective trial are probably related to differences in study design and study population. The measurements in the study by Baumann et al. were obtained from three different cohorts, which make a bias in patient selection more likely. Furthermore, the BPD rate was 36.6%, which is much higher than in our prospective study (11.3%).

A prospective, observational study including preterm infants of 28–34 weeks GA assessed CT-proET-1 on DOL 3 and resulted in BPD prediction with a sensitivity of 88.2% and specificity of 61.5%, when a cut-off value for CT-proET-1 of 302.7 ng/L was used.^36^ In light of lower chronological age at blood sampling, inclusion of newborns with higher GA, and a remarkably high BPD rate of 24.6% in the cited study compared to 11.4% in our study, these results seem to be not directly comparable. However, they support the hypothesis that CT-proET-1 can serve as biomarker for early BPD prediction.

Several attempts at establishing clinical prediction tools for BPD development have been made in recent years, but models typically have limited positive and negative predictive values,^9^ making biomarkers promising parameters to improve the diagnostic utility of such models in order to potentially improve clinical care. Results from our study confirm that MR-proANP and CT-proET-1 are associated with the duration of supplemental oxygen and development of BPD or death in very preterm infants. However, this relationship did not persist in multivariable analyses. Therefore, at this stage, analysis of MR-proANP and CT-proET-1 appear to have limited clinical value as sole predictors of respiratory morbidity but could be useful as surrogate markers of physiological immaturity and/or prematurity-related lung disease.

Strengths of our study include the prospective design, the high number of included infants in line with the power calculation, and the structured BPD assessment at 36 weeks GA in all patients. Limitations include the low rate of infants with moderate or severe BPD in our study, and the Swiss population in general,^37^ which makes generalization of results challenging.

In summary, our study showed that MR-proANP and CT-proET-1, measured at the age of 1 week, are associated in univariable analyses with the duration of supplemental oxygen and the combined outcome of BPD or death in VLGA infants. However, their additional value for BPD prediction compared to models based solely on clinical risk factors seems to be limited.

## Supplementary information

Supplementary information
